# Positive allosteric modulator selective for adult muscle nicotinic acetylcholine receptor

**DOI:** 10.1073/pnas.2504146123

**Published:** 2026-06-02

**Authors:** Richard G. Webster, Setareh Alabaf, Anna Li, Rene Beerli, Sandra Siehler, Pierre-Eloi Imbert, Leonard Lee, Judith Cossins, Magdalena Koziczak-Holbro, Ludivine Flotte, Laurent Gaudet, Nicole Gerwin, David Beeson, Yin Y. Dong

**Affiliations:** ^a^ Nuffield Department of Clinical Neurosciences, University of Oxford, Oxford OX3 9DS, United Kingdom; ^b^Novartis Biomedical Research, Basel CH-4056, Switzerland

**Keywords:** acetylcholine receptor, allosteric modulator, myasthenia, sarcopenia

## Abstract

The muscle nicotinic acetylcholine receptor (AChR) is the key mediator of neuromuscular signal transmission and is essential for all voluntary movement in our body. In this study, we present DC-98-LC74, a positive allosteric modulator (PAM) for the adult skeletal muscle-type AChR. Through using Ca^2+^ fluorometric imaging plate reader (FLIPR) assays, we demonstrate that it is selective for the adult skeletal muscle AChR over neuronal subtypes. Neurophysiological recordings from ex vivo mouse diaphragm preparations revealed that DC-98-LC74 elongates the endplate currents of wildtype (WT) adult but not fetal channel containing diaphragms. Single channel studies on chimeric channels of the adult and fetal receptor, and in saturating concentrations of choline, suggest that the PAM does not bind at either orthosteric site, but works by increasing the unliganded open probability via a mechanism that involves the ε M2-M3 loop. We also show that DC-98-LC74 increases the burst duration of multiple fast channel mutant AChR to WT levels, suggesting that positive allosteric modulation could be a therapeutic strategy for this difficult to treat subtype of congenital myasthenia. Promising preliminary data on aged sarcopenic mice also demonstrate that positive allosteric modulation of the muscle type AChR has potential benefits not only in myasthenia but also other neuromuscular disorders involving the neuromuscular junction.

## Significance

This paper presents the finding and initial characterization of a positive allosteric modulator (PAM), DC-98-LC74, that is selective for adult muscle acetylcholine receptor (AChR). Our data indicate that DC-98-LC74 increases the resting open probability, increasing the opening times of fast channel congenital myasthenic syndrome mutants to wildtype (WT) levels, and improved nerve-induced muscle force in isolated sarcopenic mouse muscle. This suggests that positive allosteric modulation of muscle AChR could be a useful therapeutic strategy for treating neuromuscular diseases.

The muscle nicotinic acetylcholine receptor (AChR) is the key functional element in neuromuscular transmission; its kinetic function and precise location are fundamental to essential contraction of skeletal muscle. Impairment of muscle AChR function, either caused by mutations in AChR genes or autoantibodies, are the main causes of congenital myasthenic syndromes (CMS) (1) and myasthenia gravis (MG), respectively. Moreover, impaired NMJ neurotransmission is a part of the pathology of many neuromuscular diseases such as amyotrophic lateral sclerosis (ALS), spinal muscular atrophy (SMA), and sarcopenia. Consequently, therapies that improve AChR function have potential for treating a range of neuromuscular diseases.

Muscle AChR is the archetypal pentameric ligand-gated ion channel (pLGIC) that has been at the forefront of novel insights since the concept of protein receptors existed, and has been the go-to receptor ion channel for many significant discoveries of function (2). One such development was the concept of the allosteric protein receptor and the M-W-C allosteric model (3). The basis of this concept is that the protein ion channel has stable conformations (open, closed, and desensitized) separated by energy barriers. If an agonist e.g., acetylcholine (ACh) has a higher affinity at the orthosteric agonist binding site for the open vs. the closed state, in the presence of that agonist the channel will be stabilized at the open state. Similarly, if a modulator binds to another site with a higher affinity for the open vs. the closed conformational state this will also promote channel opening and enhance opening in the presence of the orthosteric agonist (4). As such allosteric modulators have been useful tools in helping our understanding of pLGIC signaling mechanisms (4) and their structure–function relationship (5).

Allosteric modulators have some distinct advantages for the pharmacological intervention of ion channel function over agonists or antagonists which are restricted by the presence of natural ligand. This means they will only enhance the inherent stimulation pattern both temporally and locationally and would likely have a more specific mode of action and better tolerability. This is exemplified by the effectiveness of positive allosteric modulators (PAMs) against other pLGIC channels, like benzodiazepines, as frontline drugs. Considerable efforts have been made to find PAMs that target AChR, and several have been identified that modify the function of neuronal AChR (e.g., α7), but to date most of these modulators have no effect on the muscle AChR (6). Saito et al. recently published the first PAMs for muscle AChR (7), and showed that the PAMs increased muscle strength in a rat model of myasthenia gravis. This demonstrated that positive allosteric modulation of muscle AChR function has potential as a therapeutic strategy for treating myasthenic syndromes. However, no data on the selectivity of these compounds or their mechanism of action were presented.

In this study we describe the finding of a PAM that is selective for adult muscle AChR, DC-98-LC74, and its action on muscle AChR current at the NMJ with detailed functional characterization at the single channel level. We present functional studies on chimeric channels to narrow down its potential binding site. We demonstrate that DC-98-LC74 (shortened to DC-98 from now on) improves the kinetics of mutant AChR that cause fast channel myasthenic syndrome (FCMS), where activations of AChR are abnormally abbreviated. Many FCMS mutations cause a severe form of CMS that responds poorly to current treatments, and is life-threatening in many cases (8). Furthermore, we present the effects of DC-98 on neuromuscular transmission in ex vivo mouse diaphragm preparations from adult WT mice and a model of AChR-deficiency CMS, and on twitch force of nerve-muscle preparations from sarcopenic mice. With further investigation and development, PAMs may offer an alternative therapeutic strategy for treating myasthenic syndromes and other neuromuscular disorders with abnormalities in neuromuscular transmission.

## Results

### Identification of AChR PAM DC-98.

**Fig. 1.**
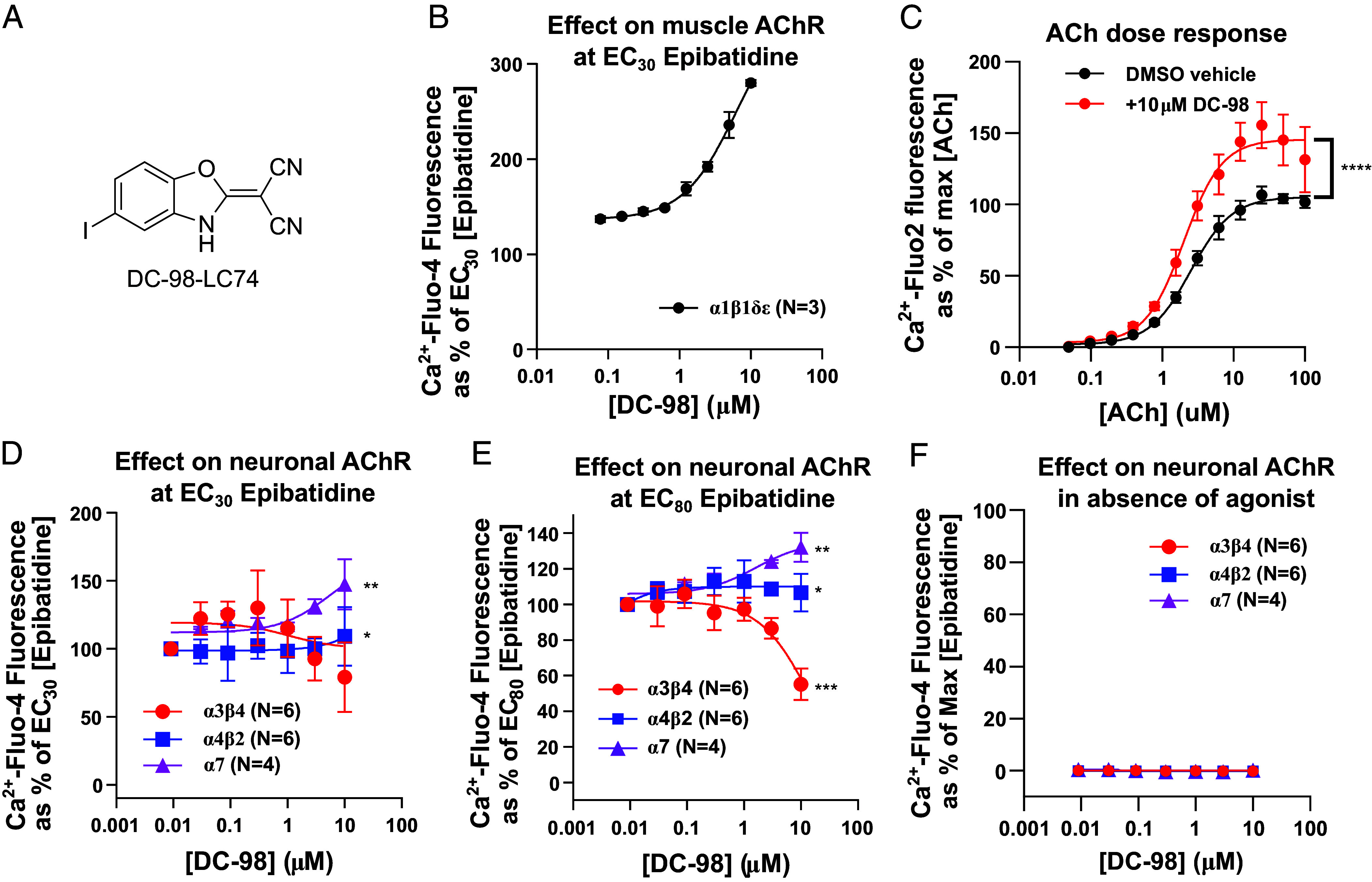
Effect of DC-98 on different subtypes of AChR. Ca^2+^ FLIPR assays were used to assess the effects of DC-98 on different subtypes of AChR. (*A*) Structure of DC-98. (*B*) Dose–response curve of DC-98 on adult muscle type AChR activity in the presence of EC_30_ [Epibatidine] (N = 3), showing a dose-dependent positive effect. Fitted using [Agonist] vs. response—variable slope (four parameters) to see if the data fitted with a stimulatory dose–response curve. The data fitted well, with an R^2^ = 0.9882, maximum effect = 367.1, 310.6-737.0 95% CI, EC_50_ = 6.5, 4.0-42.0 95% CI. (*C*) Effect of 10 µM DC-98 on adult muscle type AChR dose–response curve for ACh, showing that only positive effects are observed at all concentrations (N = 3). Fitted using [Agonist] vs. response—variable slope (four parameters) constraint: EC_50_ > 0. vehicle: R^2^ = 0.9915, EC_50_ = 2.520, 2.259-2.811 95% CI, Maximum fluorescence = 105.4, 102.2-108.7 95% CI; +10 µM DC-98: R^2^ = 0.9653, EC_50_ = 2.019, 1.620-2.501 95% CI, maximum fluorescence = 145.6, 137.7-154.2 95% CI. Comparisons of curves were carried out with a sum of squares F test to see if addition of 10 µM DC-98 produced a significant difference to vehicle, which it did, increasing the maximum fluorescence significantly (*P* < 0.0001), but not the EC_50_ (*P* = 0.1180) (*D* and *E*) DC-98 dose–response curves on neuronal subtypes of AChR in the presence of EC_30_ & EC_80_ [Epibatidine] respectively (N = 4-6). (*D*) Fitted using [Agonist] vs. response (three parameters), constrained by Top > 0, comparing fits to see whether the maximum effect (Top value) of each curve deviated significantly from 100, i.e., whether DC-98 had a significant difference from baseline. A significant deviation was observed on α4β2 (*P* = 0.0242, R^2^ = 0.7889)) and α7 (*P* = 0.0080, R^2^ = 8170), but not on α3β4 (*P* = 0.1082, R^2^ = 0.6960). However, no reliable values could be calculated for EC_50_ or the maximum effect for any receptor subtype, i.e., 95% CI could not be defined. (*E*) Fitted using [Inhibitor] vs. response (three parameters), constrained by Bottom > 0, comparing fits to see whether they deviated significantly from the baseline value of 100. Data for all three receptor subtypes deviated significantly—α3β4 (*P* = 0.0006, R^2^ = 0.9532), α4β2 (*P* = 0.0415, R^2^ = 0.6877), and α7 (*P* = 0.0026, R^2^ = 0.8957). An IC_50_ value of 13.45 (3.283-22.04 95% CI) was calculated for α3β4, but could not be calculated for the α4β2 or α7 subtypes. No stable maximum effect could be calculated for any receptor subtype. (*F*) DC-98 dose–response curves on neuronal subtypes of AChR in the absence of any orthosteric agonist, presented as a percentage of that elicited by 5 mM epibatidine (N = 4-6). Fitted using [Agonist] vs. response (three parameters), constrained by EC_50_> 0, comparing fits to see whether the Top value of each curve deviated significantly from 0. None of the datasets deviated significantly from 0 (α3β4: *P* = 0.1981, R^2^ = 0.9054; α4β2: *P* = 0.8253, R^2^ = 0.6492; and α7: *P* = 0.1474, R^2^ = 0.5485). All error bars shown represent SDs of the mean.

To identify a positive allosteric modulator of muscle type AChR an unbiased high-throughput screen of the Novartis compound archive, comprising more than 1.8 million structurally highly diverse compounds was conducted. Compounds were screened at 10 μM using Ca^2+^ FLIPR assays on HEK293 cells that express adult muscle AChRs (α1β1δε), and was performed with submaximal effective concentrations of the orthosteric agonist epibatidine (170 nM, corresponding to its EC_30_ concentration, *SI Appendix*, Fig. S1*A*). In this way, compounds that produced PAM and agonist effects were identified. These hits were then counterscreened in the absence of epibatidine. Any compounds that exhibited a response in the absence of epibatidine, i.e., had agonist properties, were subsequently excluded. This led to the identification of DC-98 (Fig. 1*A* depicts the structure of DC-98), which exhibited a remarkable positive allosteric effect on muscle AChR in the initial screening Ca^2+^ FLIPR assays (Fig. 1*B*). Further ACh dose–response curves in CN21-CHRNG knockout cells, which were engineered from human rhabdomyosarcoma cells that stably express adult human muscle AChR (*SI Appendix*, Fig. S1 *C* and *D*) (9), in the presence of 10 µM DC-98 or DMSO vehicle showed that DC-98 significantly increased the maximum effect of ACh stimulation (145.6% with DC-98, 137.7 to 154.2% 95% CI, vs. 105.4% with vehicle, 102.2 to 108.7% 95% CI, *P* < 0.0001), with no antagonistic effects at any ACh concentration (Fig. 1*C*).

### Selectivity of DC-98 for Different Types of AChR.

The selectivity of DC-98 for different subtypes of AChR was also assessed by Ca^2+^ FLIPR assays in HEK293 cells transfected with α7, α3β4, or α4β2 AChR using very similar conditions as for α1β1δε. Different concentrations of epibatidine were used to help reveal PAM ([EC_30_], Fig. 1*D*) antagonist ([EC_80_], Fig. 1*E*), and agonist (no epibatidine Fig. 1*F*) properties (see *SI Appendix*, Fig. S1*B* for epibatidine dose–response curves for each receptor subtype). A significant PAM effect was observed on the α7 (*P* = 0.0080) and α4β2 (*P* = 0.0242) receptor subtypes, but the effects are too small to estimate an EC_50_ or maximal effect (Fig. 1*D*). An inhibitory effect was observed on α3β4 AChR, with an estimated IC_50_ of 13.45 µM (3.283 to 22.04 µM 95% CI); but no maximum effect could be reliably calculated (Fig. 1*E*). No significant agonist effects were observed for any receptor subtype (Fig. 1*F*). Comparing the effects of the DC-98 on the different receptor subtypes at 10 µM using one-way ANOVA, Turkey’s multiple comparisons test, it had a significantly greater effect on the α1β1δε receptor (280 ± 3.5%) compared to all other subtypes (147.34 ± 18.43% for α7, 109.20 ± 21.70% for α4β2, and 79.1 ± 25.67 for α3β4, *P* < 0.0001).

### DC-98 Increases Burst Duration of Adult Wildtype and Fast Channel AChR.

**Fig. 2.**
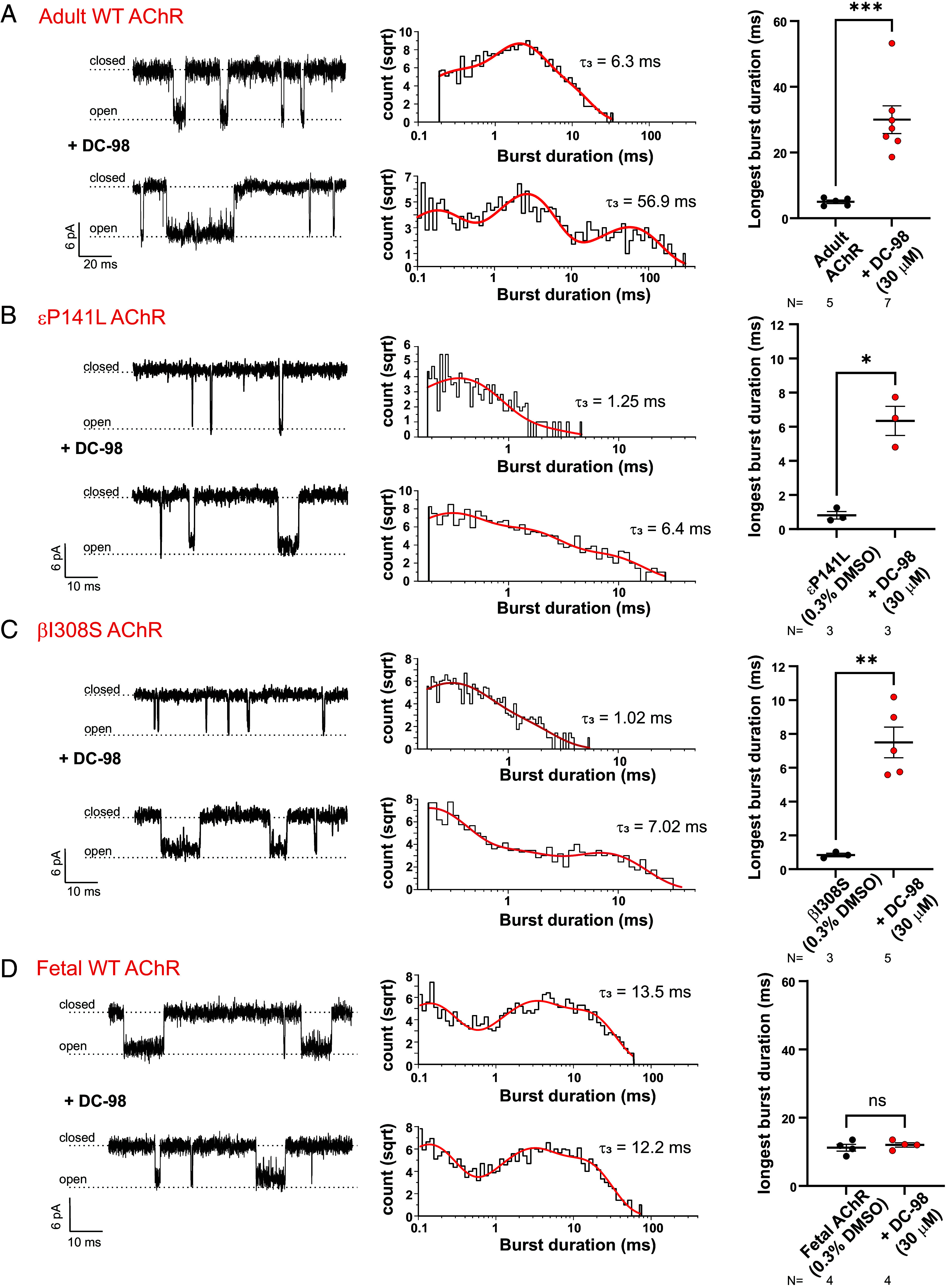
DC-98 prolongs AChR burst duration in adult WT and Fast channel mutants. Each panel shows example traces of single channel recordings of adult muscle AChR (*Left*) expressed by transfected HEK293 cells. Channel openings are downward deflections, recorded at +80 mV pipette potential. Burst duration histograms are presented in each panel (*Center*) with τ3 indicated. Graphs presented in each panel (*Right*) compare the mean (±SEM) τ3 recorded for individual patches in the presence of 0.3% DMSO vehicle control or 30 µM DC-98 in 0.3% DMSO. (*A*) shows adult WT AChR recorded with 100 nM ACh ± 30 µM DC-98 in the pipette (N = 5 and 7). (*B*) shows ɛP141L fast channel mutant AChR recordings with 500 nM ACh ± 30 µM DC-98 in the pipette (N = 3). (*C*) shows βI308S fast channel mutant AChR with 500 nM ACh and ± DC-98 in the pipette (N = 3 and 5). (*D*) shows fetal AChR with 100 nM ACh ± DC-98 in the pipette (N = 4). Burst duration histograms are fit by some of 3 exponentials (solid red line) with longest population time constant (τ3) displayed. *Right* aspect of each panel shows τ3 values from individual patches with mean ± SEM displayed, with either vehicle control or 30 µM DC-98, data for each channel ± 30 µM DC-98 were compared by the unpaired *t* test.

When an orthosteric agonist like ACh binds to AChR, they trigger open and closing events in bursts of activity. Missense variants that cause fast or slow channel myasthenia reduce or increase muscle AChR burst duration respectively, which is how they are usually diagnosed using single channel studies (10, 11). To test whether DC-98 alters the burst duration of adult muscle AChR, and could improve fast channel kinetics, single channel activity was recorded with DMSO control or DC-98 added to the pipette solution (in addition to ACh at 100 nM for adult WT and 500 nM for εP141L and βI308S) in transfected HEK293 cells. For WT AChR, the population of longest bursts recorded had a mean exponential decay time (tau) of 5.02 ± 0.52 ms (n = 5), which is typical of muscle AChR with 100 nM ACh applied via the patch pipette (12). Addition of 30 µM DC-98 to the pipette solution led to a prolongation of recorded bursts, with mean tau increasing to 30.0 ± 4.23 ms (n = 7) (Fig. 2*A*). DC-98 was further tested on two known fast channel mutant AChR (ɛ Pro141Leu & β Ile308Ser) observed in FCMS patients (8), with 500 nM ACh in the pipette. In each case the longest burst of activity had a mean tau of 0.81 ± 0.27 and 0.84 ± 0.1 ms (n = 3) respectively in the absence of DC-98, typical of fast channel mutant AChR. With the addition of DC-98 (30 µM), mean tau duration was significantly prolonged to 6.34 ± 0.85 (n = 3) and 7.50 ± 0.90 ms respectively (n = 5) (Fig. 2 *B* and *C*). Finally, we tested the effect of DC-98 on WT γ containing fetal receptors using the same experimental parameters as WT adult AChR, and saw no significant increase in burst duration in the presence of DC-98 vs. DMSO control (Fig. 2*D*). Transfected HEK293 cells patched with DC-98 alone in patch pipettes elicited no observable channel activity.

### DC-98 Increases Open Probability of Clustered AChR Activity.

**Fig. 3.**
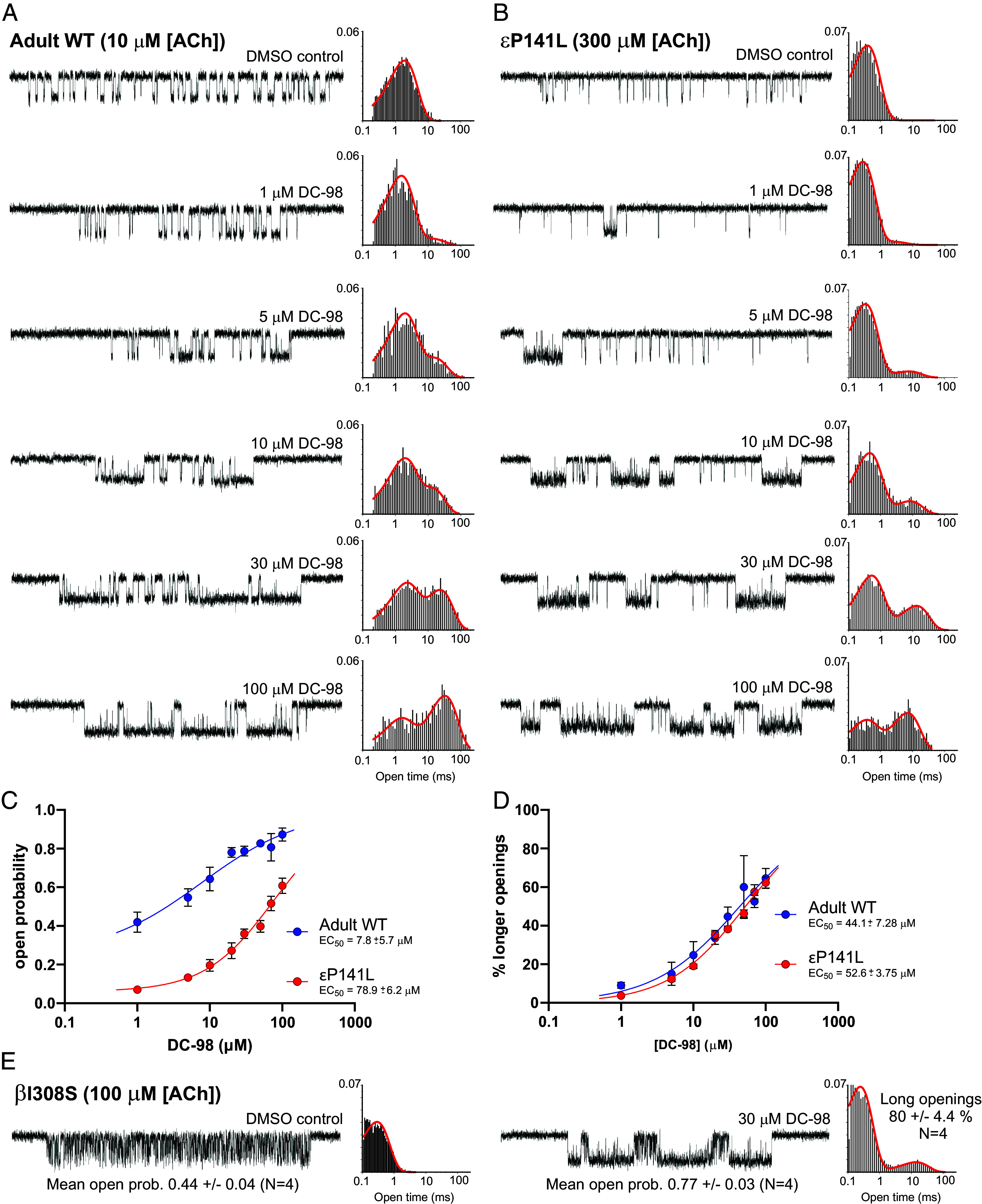
DC-98 increases open duration and proportion of long openings within clusters of activity in a dose-dependent manner. Single channel clusters of activity data for adult WT AChR and εP141L AChR respectively are shown in (*A* and *B*) respectively. Example traces of 250 ms single channel recordings are shown on the *Left* (downward deflections are channel openings). Example histograms of all openings from all clusters observed in an individual patch are shown on the *Right*, where proportion of channel open events are plotted against the open duration. In the patching pipette solution 10 µM ACh was added for WT AChR, and 300 µM ACh was added for εP141L AChR, along with varying [DC-98] or 1% DMSO vehicle control ([DMSO] is constant at 1% for all DC-98 concentrations). In the example histograms, the red line indicates maximum-interval-likelihood (MIL) fit of either 1 or 2 populations of openings. (*C* and *D*) show plots of open probability and % of longer openings within defined clusters vs. DC-98 concentration for adult WT or εP141L AChR activity respectively. Each symbol is the mean (±SEM) of N = 3-7 patches per concentration, lines indicate fit to 3 parameter Logistic function (maximum constrained to 1 and 100 for *C* and *D* respectively) to derive EC_50_ values. EC_50_ values were compared by sum of squares F-test, 280 (*P* < 0.001) and 1.43 (*P* = 0.282), respectively. (*E*) shows example traces and open duration histogram plots of βI308S AChR cluster activity at 100 µM ACh with either 1% DMSO control or 30 µM DC-98 in 1% DMSO, histogram plots show open durations within clusters. Red lines indicate fit for either 1 or 2 populations. Mean open probability was compared by the unpaired *t* test *P* < 0.001.

To determine the apparent efficacy of DC-98 to modify AChR function, recordings were made at a concentration of ACh (10 µM for Adult WT, Fig. 3*A*, and 300 µM for εP141L, Fig. 3*B*) that elicits clusters of channel activity from a single channel separated by periods of channel desensitization (Fig. 3). This allows examination of the open probability within an individual cluster and the behavior of a single channel. DMSO vehicle was held constant during these recordings (1%) while increasing DC-98 concentrations from 1 to 100 µM (limited by DC-98 solubility) was added to the pipette in addition to ACh. With vehicle alone, clusters of activity had a mean open probability of 0.31 ± 0.02 (n = 3) for adult WT and 0.05 ± 0.01 (n = 5) for εP141L. With DC-98 added, the nature of channel activity changed, displaying modal activity where periods of longer openings were observed within each cluster in addition to the WT opening and closing behavior. The frequency of these longer openings observed within each cluster correlated with DC-98 concentration (Fig. 3*B*). At 100 µM, the mean open probability was 0.87 ± 0.02 (n = 4) for adult WT and 0.61 ± 0.04 (n = 7) for εP141L. Open probability analysis suggested the EC_50_ of DC-98 was 7.8 ± 5.7 µM for adult WT and 78.9 ± 6.2 µM for εP141L (Fig. 3*C*), a significant ~10-fold change (*P* < 0.001) while the apparent EC_50_ for the increasing proportion of long openings did not show a significant difference between WT and εP141L channels (Fig. 3*D*, *P* = 0.282).

When DC-98 was applied to another fast channel mutant AChR (βI308S), a similar response was observed. Clusters of activity elicited by a higher concentration of ACh alone (100 µM) contained very brief openings within each cluster and a low overall open probability of 0.44 ± 0.05 (n = 4). Upon the addition of 30 µM DC-98, sustained periods of longer openings were observed, interspersed by typical activity of the fast channel, as seen continuously in vehicle controls (Fig. 3*E*). Overall, for βI308S clusters, open probability increased to 0.77 ± 0.03 (n = 4) and the proportion of longer opening increased to 80 ± 4.4%.

### DC-98 Can Increase AChR Cluster Open Probability Even When Orthosteric Binding Site Is Saturated.

**Fig. 4.**
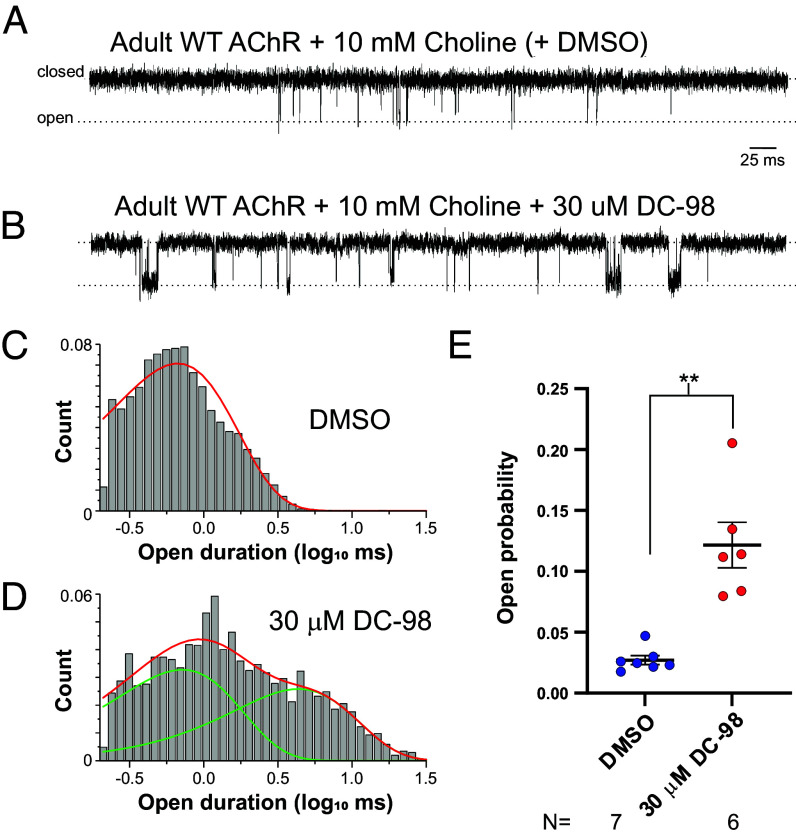
AChR open probability in saturating [Choline] is enhanced by DC-98. Example traces of WT AChR cluster activity in presence of 10 mM Choline and 0.3% DMSO (*A*) or 30 µM DC-98 + 0.3% DMSO (*B*). (*C* and *D*) Example histograms plotting total number of channel openings against open duration recorded in a single WT AChR patch in the presence of 10 mM Choline and 0.3% DMSO or 30 µM DC-98 and 0.3% DMSO. (*E*) Shows mean (±SEM) open probability of clusters, each symbol is an individual patch (N = 7 and 6 respectively. The data ± DC-98 were compared by an unpaired *t* test. In the presence of DC-98, longer openings are observed, alongside the brief openings seen in traces with Choline and DMSO alone.

For fast channel CMS mutant AChR, the addition of DC-98 appeared to increase the maximum open probability achieved for ACh. However, as was previously shown (13), high concentrations of ACh blocked WT channels with an apparent decrease in observable amplitude (13), limiting the maximum ACh concentration that could be used to study these very brief events. Therefore, to study the effect of DC-98 on channel open probability, the weak agonist choline was used to generate WT AChR activity. Even at a high [choline] of 10 mM, close to saturating dose, the maximum open probability was ~0.05 within clusters and a reduced propensity to desensitization. However, the addition of DC-98 altered these characteristics, increasing overall open probability to 0.12 ± 0.02 (n = 7-6 patches) compared to 0.03 ± 0.003 in DMSO control. Additionally, individual channel openings within clusters were prolonged. With DMSO vehicle control a single open population with a tau of 0.58 ± 0.04 ms (n = 7) was observed, however in the presence of 30 µM DC-98 two distinct populations were observed (0.71 ± 0.05 ms and 4.34 ± 0.32 ms, n = 6, Fig. 4). This suggested that the action of DC-98 was not simply an alteration of efficacy/affinity for choline at the orthosteric binding site (since this was already saturated) but altered the properties of the channel such that more activity is generated by a saturating dose of choline.

### DC-98 Increased the Duration of Endplate Currents in Adult Mouse Diaphragm, but Not a Mouse Model of AChR Deficiency That Relied on Fetal AChR.

**Fig. 5.**
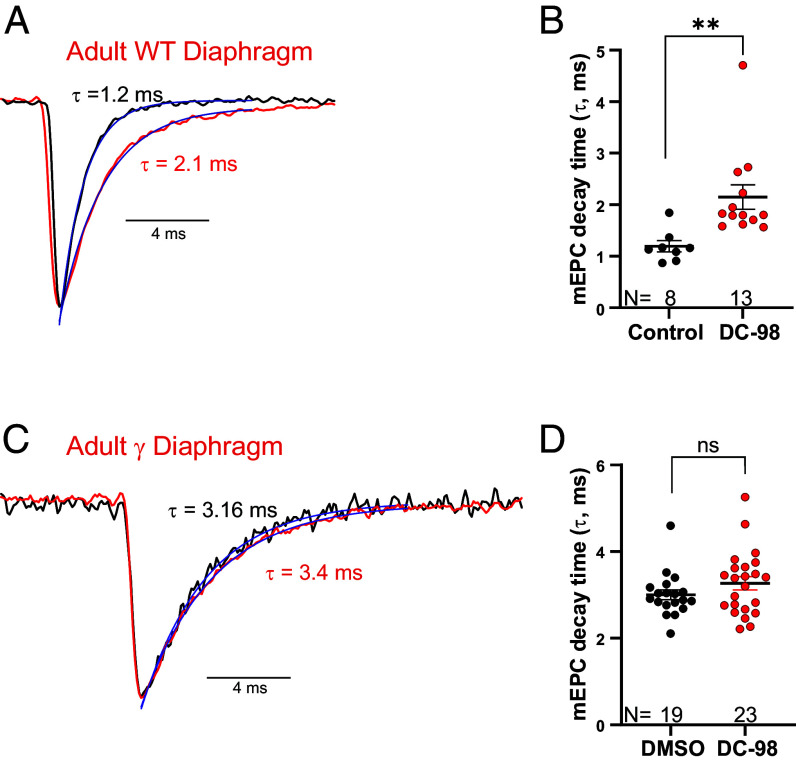
DC-98 prolongs adult WT AChR but not ɣ AChR current. (*A*) shows amplitude-normalized example traces of mEPC (averaged from detected events from a single fiber) with absence (black) or presence (red) of 30 µM DC-98. Traces are fit to single exponential decay and time constants are displayed. (*B*) panel shows exponential time constants for control or DC-98 treated adult diaphragm, panel shows mEPC decay tau recorded from adult WT diaphragm, mean ± SEM are indicated, each symbol is from an individual fiber, measured from averaged detected events (N = 8 and 13). (*C*) shows equivalent examples of mEPC recorded from ɣAChR-deficiency model diaphragm, with either DMSO vehicle control (black) or 30 µM DC-98 (red). (*D*) panel shows mEPC decay tau recorded from deficiency model diaphragm, mean ± SEM are indicated, each symbol is from an individual fiber, measured from averaged detected events (N = 19 and 23). Data ± DC-98 were compared by the unpaired *t* test.

To test whether DC-98 affected endplate currents resultant from the release of ACh from nerve terminals, we applied DC-98 in mouse phrenic nerve/diaphragm ex vivo preparations. Two-electrode voltage clamp was used to measure the kinetics of AChR currents in muscle fibers. Miniature endplate currents (mEPC) were recorded, which are currents produced by the opening of postsynaptic AChR in response to ACh from spontaneous released singular presynaptic ACh-containing vesicles. The duration of the mEPC is determined by the kinetics of AChR, since rapid removal of ACh from the synapse prevents multiple activations of AChR or desensitization. In adult WT preparations mEPC are significantly prolonged by 30 µM DC-98, with mEPC decay tau increased from 1.19 ± 0.11 (pretreatment) to 2.15 ± 0.24 ms in 8 and 13 fibers from 2 preparations each (Fig. 5 *A* and *B*). We also tested DC-98 in diaphragms from a Chrne-deficiency mouse model that expresses γ subunit containing fetal AChR exclusively (14), as no mouse model of fast channel CMS exists. Since weakness in this model is caused by insufficient endplate depolarization, a prolongation of AChR burst duration would help mitigate the deficient depolarization. In these preparations, mEPC was not prolonged significantly, by 30 µM DC-98, with control mEPC decay tau (0.3% DMSO) averaging 3.04 ± 0.12 ms vs. 3.20 ± 0.19 ms with 30 µM DC-98 added (n = 3 preps) (Fig. 5 *C* and *D*). Unexpectedly, changes in input resistance were seen in response to DC-98. In diaphragm preparations from WT mice, input resistance increased from 0.72 ± 0.05 to 1.17 ± 0.10 MΩ (n = 9 and 13 fibers, respectively, *P* = 0.001). In diaphragm preparations from AChR-deficiency mice, the change in input resistance was more pronounced, increasing from 1.03 ± 0.04 to 1.67 ± 0.08 MΩ (n = 26 and 24 fibers, respectively, *P* < 0.0001), DMSO did not affect input resistance (0.92 ± 0.05 MΩ, n = 21 fibers; *P* = 0.38 vs. untreated), see *SI Appendix*, Fig. S4.

Preliminary characterization of the effects of DC-98 in diaphragm preparations using single electrode recordings of evoked neurotransmission was also carried out. They revealed a more complex action of DC-98 in this more physiological system than would be predicted from a simple alteration of AChR channel kinetics. More differences were also observed comparing the response in adult WT and AChR-deficiency (Def) preparations (*SI Appendix*, Figs. S2 and S3). Most notably, DC-98 increased mEPP halftimes in both adult WT and AChR-deficiency preps, but the response in WT was significantly greater (*SI Appendix*, Fig. S2*C*), and EPP halftime was only increased in adult WT preps (*SI Appendix*, Fig. S2*F*). mEPP frequency was significantly and equally increased in both adult WT and AChR-deficiency preps (*SI Appendix*, Fig. S2*B*), while quantal content (QC, the number of vesicles released per stimulation) was only increased in adult WT (*SI Appendix*, Fig. S2*E*). In both adult WT and AChR-deficiency preparations mEPP amplitudes were increased, with a significantly larger change in AChR-deficiency preps (*SI Appendix*, Fig. S2*A*). This may be related to changes observed in input resistance since mEPC amplitude did not increase in either adult WT or AChR-deficiency preparations (*SI Appendix*, Fig. S4 *A* and *D*). The small increase seen in AChR-Def preps is likely a DMSO effect on mEPC amplitude and not DC-98-related (-1.00 ± 0.05; n = 19 fibers, *P* = 0.06 vs. untreated and *P* = >0.99 vs. DC-98 treated) (*SI Appendix*, Fig. S4*D*). An explanation for the change in mEPP amplitude without change in mEPC amplitude, alongside the observed changes in input resistance, could be off-target inhibition of potassium or chloride background currents. This might also underlie changes in mEPP frequency observed.

In 40% of fibers (19/47 fibers, n = 5 preps) from WT mice, a large, disordered burst of mEPPs was observed directly following initial phrenic nerve stimulation, this continued for 3 or 4 stimuli and then reverted to a normal but prolonged EPP. This response was observed in at least 1 fiber from every preparation studied, an example of which is shown in *SI Appendix*, Fig. S3 *C* and *D*. This phenomenon was never observed in AChR-deficiency preparations (*SI Appendix*, Fig. S3 *A* and *B*, n = 6 preps, n = 52 fibers).

### Effect of DC-98 on Chimeric AChR.

**Fig. 6.**
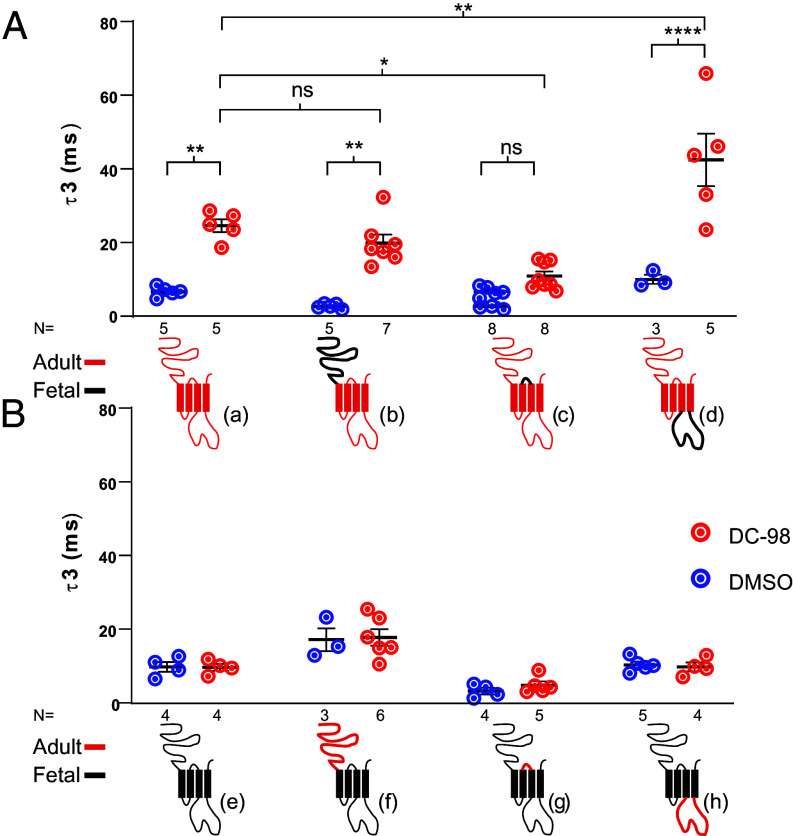
Adult ɛ and fetal ɣ AChR subunit chimera identify region mediating sensitivity to DC-98. (*A* and *B*) shows τ3 for longest burst population for various AChR constructs (as indicated by simplified pictograms) with either vehicle control or 30 µM DC-98 (+100 nM ACh). Each symbol is an individual recording with mean ± SEM indicated for each group. Pictograms signify which regions of adult or fetal AChR have been swapped. (*A*) shows data from predominant adult AChR (red) with inserted gamma subunit regions (black) (mutants a, b, c, and d). (*B*) shows data from predominant fetal ɣ AChR (black) with inserted ɛ subunit regions [red (mutants e, f, g, and h)]. Each symbol plotted represents data from an individual patch, mean ± SEM is also indicated (N = 3-8, as indicated alongside each construct). Data for each construct ± DC-98 were compared by the unpaired *t* test. All comparisons for data in panel *B* were nonsignificant.

As DC-98 only seems to affect ε containing adult muscle AChR, but not γ containing fetal AChR, we constructed chimera of ε and γ to identify potentially key regions for DC-98 binding and activity. Regions of the ε subunit were exchanged for equivalent regions of the γ subunit, and tested to see if DC-98 reactivity was abrogated. We first tried exchanging either the large (N-terminal) extracellular or intracellular regions (M3-M4 loop) of ε subunit for γ-containing equivalent regions (Fig. 6*A* mutants b&d). The effect of DC-98 remained unchanged in the ε subunit with γ N-terminal extracellular domain (residues 1 to 239) with a mean τ3 of 19.8 ± 2.3 ms as compared to 24.6 ± 1.7 ms in the wildtype adult AChR in the presence of DC-98, suggesting no significant involvement of the ε subunit N-terminal extracellular domain in DC-98 binding. Adding 0.3% DMSO vehicle control to the ε(γN-terminal EC domain) led to a shortening of τ3 as compared to WT adult AChR (2.7 ± 0.3 ms vs. 6.7 ± 0.6 ms), *P* < 0.001. Swapping the intracellular M3-M4 loop of ε-subunit for γ-containing equivalent region resulted in significantly increased τ3 from 9.97 ± 1.2 ms with DMSO vehicle control to 47.33 ± 7.6 ms with DC-98, indicating a preservation of the effects of DC-98 suggesting this region is not involved in the DC-98 effect. Intriguingly, exchanging the juxta-membrane M2-M3 region of the adult channel for the fetal sequence (Fig. 6*A* mutant c) significantly reduced the effect of DC-98, with τ3 of 24.6 ± 1.7 ms in wildtype adult AChR to 10.9 ± 1.3 ms, giving no significant increase over the DMSO only control. This suggested that this region could be involved in DC-98 binding and is important to its mechanism of action. Although γ containing ε M2-M3 loop gave the channel more adult-like kinetic features such as shorter burst duration (3.22 ± 0.9 ms), the burst elongating features of DC-98 were not seen when the compound was added (τ3 remained at 4.78 ± 1.1 ms). Swapping the extracellular, intracellular, or juxta-membrane M2-M3 regions of predominant fetal γ AChR with equivalent ε subunit regions (Fig. 6*B* mutants f, g, h) did not confer sensitivity to DC-98.

### DC-98 Improved Nerve-Induced Muscle Force in Isolated Sarcopenic Mouse Muscle.

**Fig. 7.**
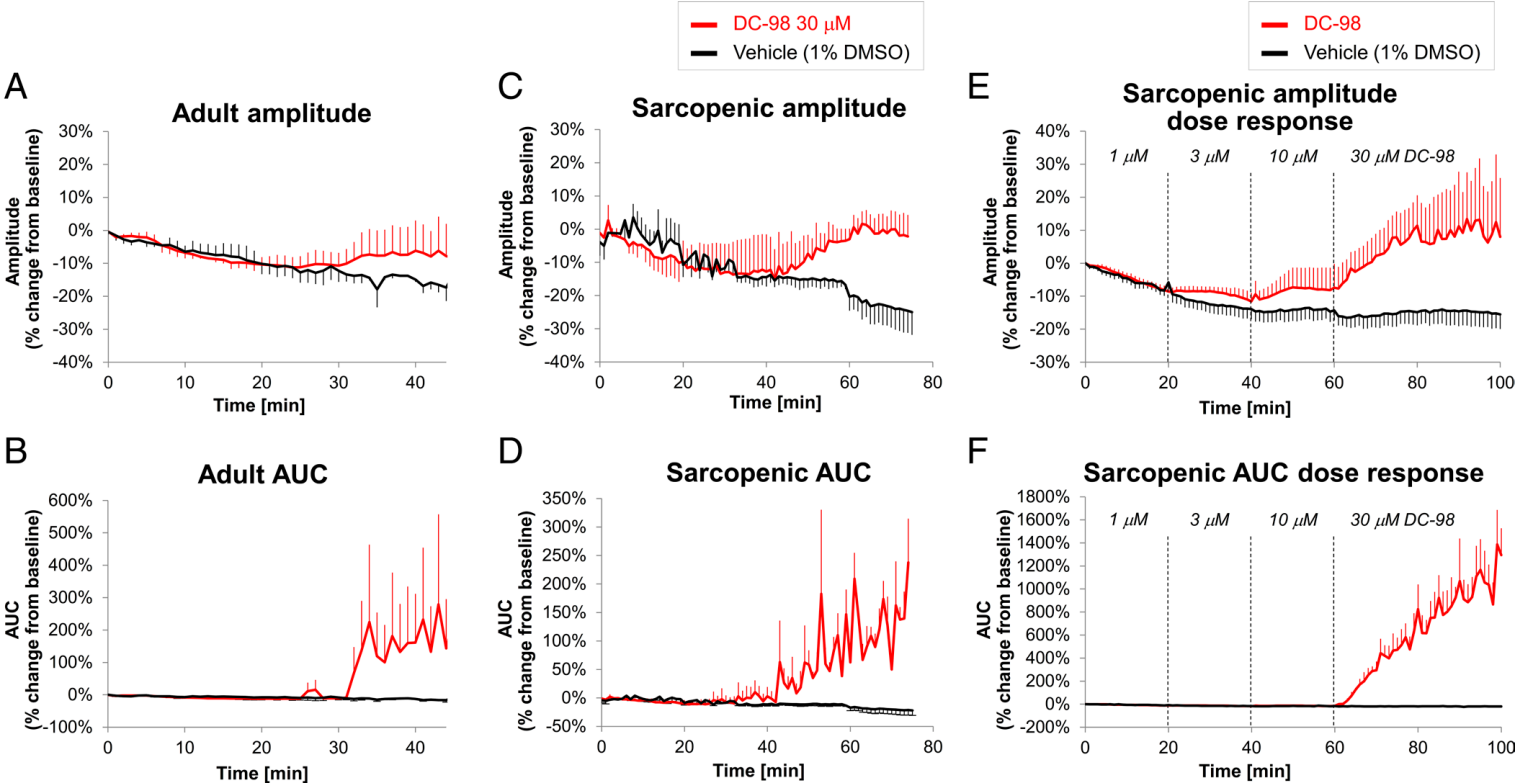
DC-98 reverses muscle fatigue and prolongs muscle twitches in isolated nerve-muscle preparations of sarcopenic mice. (*A*–*D*). Treatment of field stimulated nerve-muscle preparations from adult mice (*A* and *B*) with either 30 µM DC-98 (n = 2) or vehicle (1% DMSO) (n = 2) and sarcopenic mice (*C* and *D*) with either 30 µM DC-98 (n = 3) or vehicle (1% DMSO) (n = 3). (*A* and *C*) Muscle force was measured as twitch amplitudes, and (*B* and *D*) AUC as measure of twitch amplitude and duration. (*E* and *F*) Effects of treatment with escalating doses of 1, 3, 10, and 30 µM DC-98 (n = 4) or vehicle (1% DMSO) (n = 12) on nerve-stimulated nerve muscle preparations from sarcopenic mice shown as muscle twitch amplitude (*E*) and AUC (*F*).

Disruption of action potential signal transmission at NMJs is thought to contribute to age-related loss of muscle strength and mass in sarcopenia (reviewed by ref. 15). To investigate whether the DC-98-induced prolongation of AChR burst duration and opening time translates to an improvement in NMJ transmission and muscle force, isometric muscle force was measured in nerve-extensor digitalis longus (EDL) muscle preparations of healthy adult and aged sarcopenic mice. Field-stimulation over 45 to 75 min resulted in fatigue, measured as reduction in muscle twitch amplitude, in sarcopenic (25% after 74 min) as well as healthy adult (16% after 44 min) mouse muscle preparations (Fig. 7 *A* and *C*). DC-98 treatment reversed the fatigue-associated twitch amplitude reduction fully in sarcopenic and about half (8% after 44 min) in adult muscle. DC-98-induced twitch amplitude recovery started later and took longer to plateau in sarcopenic (from 46 to 65 min) as compared to healthy adult muscle (from 32 to 40 min). Furthermore, DC-98 treatment increased muscle twitch areas under the curve (AUC) in sarcopenic as well as adult muscle (Fig. 7 *B* and *D*) by 180% and 160%, respectively, indicating that DC-98 prolongs each muscle twitch besides increasing its amplitude. Next, the effects of DC-98 treatment on NMJ transmission in nerve-stimulated sarcopenic muscle preparations were investigated (Fig. 7 *E* and *F*). DC-98 dose-dependently increased nerve-stimulated muscle twitch amplitudes by up to 28%. Interestingly, DC-98 had a much more pronounced effect on nerve-induced vs. field-induced muscle twitch AUC, i.e., muscle twitch prolongation, increasing it up to 1,406%, but only at the highest dose tested (30 µM).

## Discussion

Obtaining sufficient muscle AChR activation upon ACh release is key to neuromuscular transmission and the depolarization of skeletal muscle that instigates contraction. A large safety factor/functional reserve is necessary for the neuromuscular junction to be reliable and capable of repeat stimulation at high frequency. Impaired neuromuscular junction function is the cause of myasthenias and is also thought to be involved in the pathogenesis of other neuromuscular disorders such as sarcopenia, spinal muscular atrophy, certain myopathies, and ALS. Saito et al. (7) recently published the first reported PAMs against muscle AChR, demonstrating improved strength in a rat model of myasthenia gravis, though data were not presented on the selectivity of the compounds, or their mechanism of action.

We present DC-98-LC74, a positive allosteric modulator that is selective, but not specific, for the adult muscle AChR, having smaller, but noticeable effects on neuronal AChR subtypes, and no observable activity on the human fetal muscle type receptor. Single channel studies showed that DC-98 increased the burst duration of AChR activity and increased the open probability within clusters of activity of both wildtype receptors and FCMS mutants located in either the β or the ɛ subunits, in a dose-dependent manner. No AChR activity was observed in the absence of orthosteric agonists suggesting that DC-98 acts via an allosteric mechanism.

The activity on the ɛP141L mutant AChR suggested that DC-98 does not bind at the αɛ orthosteric ACh binding site, as this mutation was previously shown to disrupt this binding site (16). The lack of activity on the fetal channel, which has an identical αδ orthosteric site indicated DC-98 does not function via the αδ site either. This was corroborated by the activity of DC-98 in the presence of a saturating concentration of choline, which suggested that it acts by increasing the unliganded open probability (L_o_) rather than affecting ACh binding affinity (16). The L_o_ rate constant represents an unliganded receptor’s opening energy barrier and can be influenced by many factors unrelated to binding of agonist at the orthosteric binding site. This includes membrane voltage (17, 18), amino acid sequence [including slow channel gain-of-function CMS mutations (19)] and phosphorylation (20) as well as allosteric modulators. The loss of activity in a chimera of the adult channel with the γ M2-M3 loop inserted for the equivalent ε M2-M3 loop showed that this region of the ɛ subunit is important to DC-98’s mechanism of action and may be involved in compound binding. This is intriguing as the M2-M3 loop has been shown to be a key region in transitioning between closed to open states (5, 21, 22). It is also close to the PAM binding sites recently elucidated by the Hibbs lab (23) for the α7 receptor. Therefore, a compound that selectively binds to this region when it is in a particular conformation is likely to change the stability of different conformational states and allosterically modulate the kinetics of AChR. However, high-resolution structural data are needed to determine exactly where the compound binds.

Positive allosteric modulators of pentameric ligand-gated ion channels have been used as front-line medicines for many conditions such as benzodiazepines for psychiatric disorders. Considerable efforts have been made to develop allosteric modulators of neuronal AChR such as the α7 receptor (reviewed in ref. 24). DC-98 may serve as a template for developing future drugs for myasthenia and other types of neuromuscular diseases. In particular, we show in a cell culture model system that it can reverse the effects of missense mutations on separate AChR subunits identified in CMS patients with severe fast channel syndrome that were found to respond very poorly to all currently available treatments. An AChR PAM would likely be complementary to existing treatments, typically cholinesterase inhibitors that reduce ACh breakdown and aminopyridines that increase ACh release. As Saito et al. demonstrated, PAMs for the muscle AChR could also play a role in treating myasthenia gravis (7). As a PAM that affects channel kinetics is likely to be fast acting, it could be used acutely to treat patients during a myasthenic crisis, which are life-threatening respiratory events that affect roughly a fifth of MG patients (25) and are also common in certain subtypes of CMS such as those with mutations in *RAPSN* (26) as well as FCMS (8).

We also observed that DC-98 treatment restores the nerve-induced muscle twitch amplitude and increases muscle twitch AUC profoundly in sarcopenic mouse nerve-muscle preparations. This is likely due to the increased EPP amplitude and duration, which would increase the number of muscle fibers reaching the depolarization threshold upon nerve stimulation. Off-target inhibition of background potassium or chloride currents could also contribute to enhanced muscle twitch duration. This suggests that AChR PAM treatment could potentially have broader applications, such as improving the muscle force in sarcopenia patients. Although it has been recognized that impaired NMJ transmission is a major contributor to age-related muscle weakness in sarcopenia (27), no treatment is available to date to restore neuromuscular transmission and muscle force in sarcopenia patients.

### Limitations of This Study.

Along with the on-target PAM effects of DC-98 observed, several off-target, or unpredicted effects were also observed. We saw increased mEPP frequency in both WT and gamma mouse diaphragms, suggesting that DC-98 has an unknown effect on presynaptic ACh release, that is not adult muscle AChR related. We also observed a dysregulated release of ACh after the initial EPP in a proportion of WT diaphragm fibers, but not all fibers, which was never seen in AChR-deficiency mouse diaphragms. Changes in input resistance were also observed with DC-98 treatment and could contribute to these unexpected findings, and might also contribute to increased muscle excitability and enhanced muscle twitch. These were unexpected effects that we cannot fully explain with our existing data, but the fact that some were only observed in the WT mice, suggests that it may be an adult AChR mediated mechanism, or the target is missing from the disrupted AChR-deficient NMJs. We speculate that since this ultimately affects presynaptic ACh release, for a few EPPs, after which it disappears without affecting later EPPs, it is perhaps caused by some sort of feedback mechanism triggered by the longer openings of postsynaptic AChR (28), which may cause the release of ACh from a different population of presynaptic vesicles to those normally released upon stimulation (29, 30). This vesicle pool becomes depleted after a few EPP, requires reloading, and can be mobilized again after ~30 s. These elongated EPP may also contribute to the elongated muscle twitch observed in the isolated sarcopenic muscles.

Further development of DC-98 is obviously required to improve its potency and specificity before it can be used medically. If one could isolate its presynaptic and postsynaptic effects, it may also become a useful tool to better understand different NMJ homeostatic signaling mechanisms. Once a more potent and specific PAM is developed, in vivo studies would be required to determine if an AChR PAM treatment can restore muscle force and reduce muscle fatigue sustainably in animal models of different neuromuscular diseases.

### Final Summary.

DC-98 is a PAM that is selective for the adult muscle type AChR. Our data suggest that it increases the resting open probability of the receptor, without binding to the orthosteric sites, but perhaps works via the M2-M3 loop. It increased the opening times of two different fast channel variants, in two different subunits of AChR, suggesting positive allosteric modulation could be a useful treatment strategy for these difficult to treat subtype of CMS. It also had a positive effect on twitch force in isolated muscles from sarcopenic mice. However, DC-98 also produced some unexpected effects on NMJ activity, meaning further developments to the molecule are required before in vivo use.

## Materials and Methods

### Preparation of DC-98-LC74.

To 2.00 g (8.51 mmol) 2-Amino-4-iodophenol in 5 mL DMSO were added 0.8 mL (8.5 mmol) of an aqueous solution of NaOH (32%). After stirring at RT for 1 h, 1.45 g (8.51 mmol) 2-(bis(methylthio)methylene)malononitrile was added and the reaction mixture was heated to 60 °C for 16 h. The reaction mixture was poured onto 10 mL TFA/20 mL water and the reaction flask was rinsed with 25 mL water. At RT, the light brown suspension was filtered off and washed with a small portion of water to give 2.35 g of 2-(5-iodobenzo[d]oxazol-2-yl) malononitrile (DC-98) in 85% yield and >95% purity. ESI-MS (M-1)^−^ = 307.9, ^1^H NMR (400 MHz, DMSO-*d*_6_) δ: 7.56 (s, 1H); 7.49 (d, *J* = 8.4 Hz, 1H); 7.39 (d, *J* = 8.4 Hz, 1H).

### Epibatidine Ca^2+^-FLIPR Assay.

HEK293 cells recombinantly expressing human adult muscle AChR, i.e., α1 (NM_000079), β1 (NM_000747), δ (NM_000751) and ε subunits (NM_000080) were purchased from Millipore (#CYL305255). Cells were cultured at 37 °C, 5% CO_2_, and 95% relative humidity. For passaging, cells were detached from the cell culture flask by washing with 1× PBS without Ca^2^/Mg^2+^, and brief incubation with 0.05% trypsin/0.1 mM EDTA. Cells were split every 2 to 3 d by seeding 8 × 10^6^ cells into a T500 flask to reach 80% confluence after 3 d. For the high-throughput screening (HTS), propagating cells were cultivated, harvested, and plated in 384-well assay plates. For assay implementation and small-scale screening experiments cells were manually seeded. Passages 8-22 were utilized for experiments. The quantity of cells needed to reach 70 to 80% confluency at harvesting was 6,000 cells per well seeded 48 h ahead (cell viability was between 95.2% and 100%). *Mycoplasma* tests were regularly performed using the MycoAlert® Assay kit (#LT07-318, LONZA). Ligand-induced opening of muscle AChR channel at the plasma membrane causes influx of cations. In a Na^+^-free assay buffer the flux of Ca^2+^ ions into the cytosol can be detected using the Ca^2+^-sensitive fluorescent dye Fluo-4 loaded into cells. Signals were recorded in a kinetic fashion. The assay buffer was prepared freshly at the day of the experiment. From the Ca^2+^ kinetic curves generated by the FLIPR instrument (Molecular Devices, San Jose, CA) normalized fluorescence = dF/F = [(F_max_ - F_base line_)/F_base line_] was calculated. Stimulatory concentration–response curves were analyzed by nonlinear regression curve fitting using the four parametric fitting model of the Origin 8.5 software. In screening mode, the percent modulation of compounds was calculated relative to the epibatidine EC_30_ and EC_100_ controls on each plate.

### Selectivity Screening against Neuronal Type AChR.

Ca^2+^ FLIPR assays were carried out as described above using clonal HEK293 cells transfected with α4β2, α3β4, or α7 AChR. Cells were preplated at 6,500 cells/well in plates and incubated for 2 d. Cells were washed with automated washer (ELx50, BioTek Instrument, GMBH, Luzern) with NMG buffer (HBSS; 20 mM HEPES; 0.75 mM CaCl_2_, adjusted pH to 7.4) to remove media. On the BioTek, 30 µL of 2× loading buffer (NMG buffer; 6.4 µM Fluo-4 AM; 5 mM probenecid) was loaded per well, incubated at 37 °C, 5% CO_2_ for 45 min, and washed again with NMG buffer. Cell plates were then transferred to a fluorometric imaging plate reader (FLIPR, Molecular Devices, San Jose, CA) where 10µL of increasing concentration of compound (4× final concentration, 1:3.33 serial dilution; 8 points) was added while recording fluorescence to measure agonist effect with Epibatidine as control. After 20 min incubation at room temperature 10 µL of 5× of Epibatidine EC_30_ for allosteric mode and EC_80_ for antagonist mode, freshly prepared, were added while recording fluorescence. In agonist mode, DC-98 was applied in the absence of epibatidine, and the cellular fluorescence was compared to what is produced when E_max_ epibatidine (100% agonist) is applied (3 µM starting for α7 AChR clone, 1 µM for α4β2, α3β4 AChR clones). In allosteric modulator mode, DC-98 was added along with EC_30_ epibatidine defined for each cell line from dose–response curve (DRC) (70 nM for α7 AChR clone, 5 nM for α4β2, α3β4 AChR clones). The effect of DC-98 was calculated relative to EC_max_—EC_30_ of epibatidine, which was set as 100% and baseline, respectively. In antagonist mode, DC-98 was added along with EC_80_ epibatidine defined for each cell line from DRC (200 nM for α7 AChR clone, 75 nM for α4β2 AChR clone, 15 nM for α3β4 AChR clones). The effect of DC-98 was calculated relative to EC80 of epibatidine, which was set to 100%.

### ACh Dose–Response Assay.

CN21-CHRNG knockout cells (see *SI Appendix* for how cells were made) expressing human adult muscle AChR (9) were used for this assay. They were maintained at 37 °C, 5% CO_2_, and 95% relative humidity in DMEM (D5796, Sigma-Aldrich), supplemented with 10% FBS (A5209402, Gibco), and 1% penicillin-streptomycin-amphotericin B (A5955, Sigma-Aldrich). Cells were passaged every 2 to 3 d and split when they reached 90% confluence, by washing with PBS, pH7.4 (10010023, Gibco), followed by a 3-min incubation with 0.25% trypsin (15090046, Gibco), and seeding into T75 flasks at a density of approximately 2 × 10^6^ cells.

For assays, cells were seeded into 384-well plates (781091, Greiner Bio-One) 24 h ahead of experiments at a density of 10,000/well, sealed with sealing membranes (BERM-2000, Diversified Biotech), and incubated at 37 °C, 5% CO_2_, and 95% relative humidity without lids. Before experiments, cells were allowed to equilibrate at RT for 1 h. Culture medium was then removed by gentle blotting and brief centrifugation, and cells were loaded with 40 μL of loading solution [one vial of Calcium 5 dye bulk kit (R8186, Molecular Devices) in 200 mL of HBSS (H8264, Sigma-Aldrich), supplemented with 20 mM HEPES, pH7.3 (Fisher, 10041703), and 5 μM atropine sulfate (A10236.09, Alfa Aesar)] per well. Cells were incubated at RT with loading solution for 1 h and transferred to a FLIPR Tetra, fitted with a 470-495/515-575 LED/filter combination (Molecular Devices). 10 μL of 5× compound solution in HBSS/H, pH7.3 was then added, while recording fluorescence. After a further 10 min, 10 μL of 6× ACh solution in HBSS/H, pH7.3 was added while recording fluorescence for 2 min. Normalized fluorescence was calculated from the Ca^2+^ kinetic curves as previously described and scaled relative to cellular responses to 100 M and 0 μM ACh (100% and 0%, respectively).

### Single Channel Electrophysiology.

Recordings were made from HEK 293 cells 48 h after transfection with AChR subunit cDNAs. cDNA for EGFP was included as a marker of transfection. Recordings were performed in the cell-attached patch configuration (31) at 20 to 22 °C. The cells were bathed in a solution containing either NBS: 150 mM, NaCl; 2.8 mM, KCl; 2 mM, MgCl_2_; 1 mM, CaCl_2_, 10 mM, HEPES/NaOH; 10 mM, glucose; pH7.4. (Normal bath solution, NBS) or Hi K+ solution: 142 mM KCl; 5.4 mM NaCl; 1.8 mM CaCl_2_; 1.7 mM MgCl_2_; 10 mM HEPES/KOH; 10 mM glucose; pH7.4 The pipette solution was the same as bath solution, except glucose was omitted and ACh added. As indicated DC-98 or a DMSO equivalent volume were added to pipette solution. DC-98 at 30 µM concentration contained 0.3% DMSO and was compared with vehicle controls containing 0.3% DMSO alone; except in Fig. 3 where DMSO was uniformly 1% for all DC-98 concentrations and vehicle controls. Single-channel currents were amplified with an Axopatch 200B amplifier (Molecular Devices, Sunnyvale, California), and sampled to hard disk at 100 kHz, initially filtered at 5 kHz (−3 dB, Bessel filter). Burst duration recordings were made with the pipette potential set at +80 mV. Channel transitions were detected by 50% amplitude threshold crossings (pClamp9). Bursts were defined as groups of openings separated by closed intervals longer than a critical duration (t_crit_); t_crit_ was determined for each patch using Eq. **1** (32).

[1] $$a_{1}*e^{\frac{-tcrit}{t_{1}}}=a_{2}*\left(1-e^{\frac{-tcrit}{t_{2}}}\right),$$

where *a* and *τ* indicate area and time constant for within and between bursts. Histograms of burst duration were fitted to the sum of exponentials by maximum log likelihood. Wild type AChR ((α1)_2_, β1, ε, δ subunits) were typically best fit by the sum of 3 exponential functions. Cluster open probability was recorded at [ACh] > 10 µM. These data were analyzed using QUB software (SUNY, Buffalo, USA).

### Diaphragm Electrophysiology.

Following euthanasia according to the approved procedure by the NC3R guidelines, phrenic nerve/hemi-diaphragm preparations were dissected and bathed in Krebs solution, containing 2.5 mM CaCl2 and bubbled with 95% O2/5% CO2 (14) μ-Conotoxin GIIIB (2.5 μM, Peptide Institute Inc.) was added to the bath for 30 min to block muscle contractions. The excess unbound toxin was washed out before recordings started. Recordings were made at 22 to 23 °C.

The phrenic nerve was pulled into a suction electrode, which was coupled to a pulse generator, with an associated stimulus isolation unit (GRASS instruments S48 square pulse stimulator). Recording electrodes were connected to an Axoclamp 900A amplifier (Molecular Devices). Data signals passed through a Humbug 50 Hz noise eliminator (Quest Scientific via Digitimer). Signals were continuously digitized at 10 kHz sampling rate and filtered at 2 kHz, using Axon Digidata 1322A interface, controlled by pClamp 10 software (Molecular Devices). Depolarizations at the endplate were recorded intracellularly using a single borosilicate glass micropipette electrode. Two-electrode voltage clamp (TEVC) was performed to record membrane current. Prior to voltage clamp, current was injected (6 or 10 nA) via one electrode and depolarization of the fiber monitored on the 2nd electrode, in this way muscle fiber input resistance was measured. To achieve voltage clamp, gain and lag settings were optimized for signal to noise; gain settings ranged from 1,200 to 2,200 while a constant lag setting was used. When TEVC was established (held at −80 mV), stimulated EPCs were recorded. Remnant (uncompensated) changes in membrane voltage were 1.35 ± 0.14 mV in WT preparations and 0.62 ± 0.08 mV in AChR-deficiency preparations, typically ~3.75% and 3.3% of normal EPP amplitude. Changes in membrane potential co-incident with recorded mEPC events were undetectable. Electrodes were pulled by a programmable P-97 microelectrode puller (Sutter Instruments, Novato, CA) and filled with 3 M KCl (10 to 30 MΩ). The recording electrode/s was positioned above endplate regions, as visualized by stereomicroscope (Olympus BX51WI) under micromanipulator control (Scientifica).

Impalement of individual muscle fibers adjacent to the neuromuscular junction (endplate) was indicated by fast rise time of miniature endplate potentials (mEPPs) or currents (mEPC), defined as less than 2 ms in AChR-deficiency model mice and 1.5 ms in WT. mEPC and mEPP decay time was measured on averaged traces obtained from >20 recorded events and EPC and EPP were averaged from 20 events in a 1 Hz train. mEPP and EPP amplitude were adjusted to a standardized membrane potential of –80 mV and quantal content was calculated accounting for nonlinear summation. Recordings in the presence of DC-98 were made following at least 15 min of incubation with the compound. No fibers were continuously recorded before and after the addition of DC-98.

### Nerve-Muscle Contractility.

Male C57BL/6JRj mice (Janvier) were used at either 2 to 3 mo (adult) or 27 to 30 mo of age (sarcopenic). The mice were housed in a pathogen-free facility, with 12-h light/12-h dark cycles and free access to food and water. All the manipulations were performed under a Novartis license approved by the cantonal veterinary office. For the nerve-muscle preparation, the mouse was deeply anesthetized with urethane (1.5 g/kg) injected intraperitoneally at 10 mL/kg body weight, such that there was no response to tactile stimulation. After the dissection, mice were sacrificed by cervical dislocation.

The Extensor Digitorum Longus (EDL) muscle was carefully extracted with intact tendons on both ends, which were tied to pins using a silk thread, and with or without their intact peroneal nerve separated from the tibial nerve. As soon as the muscle was excised, it was placed in a 10 mL bubbled (95% O_2_, 5% CO_2_) Tyrode buffer solution (T2397, Sigma-Aldrich) at room temperature and allowed to equilibrate for at least 30 min. The pins on each side allowed to attach the muscle horizontally between the force transducer and a micromanipulator in the exact middle of the electrodes. The muscle was manipulated very cautiously, to avoid any direct contact, either with the hands or the pliers, to prevent impairment of muscle force.

For each individual muscle placed in its own experimental reservoir bath, the optimal supramaximal stimulation condition and the optimal muscle length (at which the muscle was generating its maximal force with single pulse) were established before electrical stimulation protocols were applied. To define the supramaximal stimulation condition, single pulses at increasing voltage were delivered until the same force was obtained twice in a row, which meant that the optimal voltage plateau had been reached. For optimal length (L0) the muscle was stretched very slowly and carefully, until the twitch responses were constant in force.

For single dose compound response experiments, the muscle was stimulated by field stimulation between the two horizontal electrodes and for the cumulative compound dose–response, the muscle was stimulated through its peroneal nerve placed into a suction electrode. 10 min after start of the electrical stimulation protocol at 0.1 Hz with single square pulses of 0.1 ms or 0.2 ms width, for the field and the nerve stimulation techniques, respectively, the compound was added into the bath and then every 20 min for the cumulative dose–response protocol.

The percent relative change in single twitch amplitude and AUC from baseline (average of the 3 last single twitch responses [t0, t-1 & t-2] before compound application) were analyzed by using the PowerLab DAQ/Labchart system (ADInstruments, UK). Results are presented as means ± SEM.

### Cloning.

To swap the N-terminal extracellular domains of ε and γ subunits, new BsiWI restriction sites were created between the extracellular domain and transmembrane domains by site specific mutagenesis using QuickChange kits (Agilent Technologies—Cat No 200515) according to the manufacturer´s protocol. Mutant constructs were transformed into 10-beta *Escherichia coli* competent cells (NEB), cultured in 500 µL LB-agar for 1 h in a shaking incubator at room temperature before 100 µL plated onto agar plates containing Ampicillin (100 mg/mL) and incubated at 37 °C overnight. The following day single colonies were picked and cultured in LB broth containing Ampicillin (100 mg/mL) and incubated overnight with moderate shaking at room temperature. Plasmid DNA was extracted using the QIAprep spin miniprep kit (Qiagen), and Sanger-sequenced to confirm insertion of BsiWI restriction site. Plasmid DNAs were then digested with BsiWI (Thermo Fisher Scientific—Cat No ER0851) and HindIII (Thermo Fisher Scientific—Cat No FD0504) following the manufacturer´s instructions and ligated into the ε pc.DNA3.1/Hygro(+) and vice versa using T4 DNA ligase (Sigma-Aldrich). The reactions were carried out using 1:3 molar insert: vector ratio in 10 µL reaction mixes consisting of vector DNA, DNA insert, T4 DNA ligase, T4 DNA ligase buffer and nuclease free water. The final ligation was confirmed by Sanger sequencing and finally the BsiWI restriction site was mutated back to the wildtype sequence in CHRNE and CHRNG using the same method as for initial mutagenesis and reconfirmed by Sanger sequencing.

Chimeras where the intracellular domain between M3 and M4 are swapped between ε and γ subunits were created as described previously (33). Chimeras of ε and γ where the M2-M3 loops are swapped (amino acid positions 292 to 307) were ordered from ThermoFisher Scientific GeneArt services and confirmed by Sanger sequencing.

### Statistics.

Results are presented as means ± SEM or SD as indicated in figure legends. Student’s *t* test for paired and unpaired samples were performed to evaluate the statistical significance of differences. Where appropriate multigrouped data were tested by ANOVA followed by multiple comparisons with appropriate correction (Graphpad Prism). For Ca^2+^-FLIPR data, nonlinear regression analysis was carried out, using sum of squares to fit curves; and either [agonist] vs. response or [antagonist] vs. response, with three or four parameters used depending on which method produced the better fit. Comparisons between dose–response curves were carried out using F tests. In figures statistical significance is indicated; ns *P* ≥ 0.05; * *P* < 0.05; **P < 0.01; ***P < 0.001; ****P < 0.0001.

## Supplementary Material

Appendix 01 (PDF)

## Data Availability

Study data are included in the article and/or *SI Appendix*.
