# Supplementary material for: Positive allosteric modulator selective for adult muscle nicotinic acetylcholine receptor
Source: Proc Natl Acad Sci U S A. 2026 Jun 2;123(23):e2504146123. doi: 10.1073/pnas.2504146123 (PMC13250566; doi:10.1073/pnas.2504146123)
Supplement: Supplementary file 1 — Appendix 01 (PDF) [file pnas.2504146123.sapp.pdf]

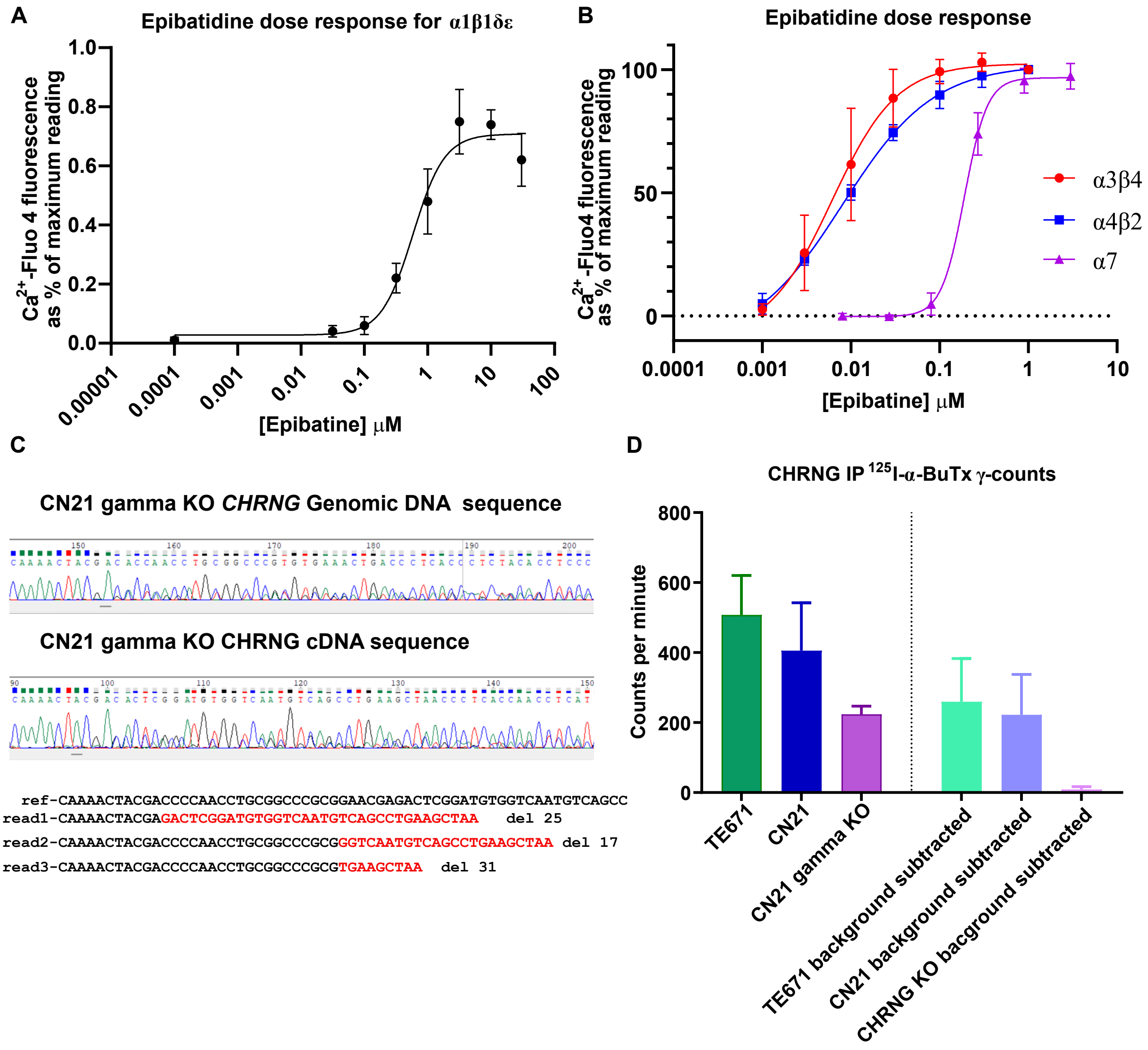

Fig. S1. Ca^{2+} FLIPR Epibatidine dose response curves. (**A**) Dose response curve from cells expressing adult muscle type AChR (N = 4). EC_{50} = 0.583 µM (**B**) Dose response curve from cells expressing neuronal subtypes of AChR (N=6 for α3β4 & α4β2, N=4 for α7). EC_{50} = 6.16 nM for α3β4, 8.06 nM for α4β2 and 0.190 µM for α7. Lines are fitted using GraphPad Prism v10.6.1 using the [Agonist] vs. response – variable slope (four parameters) method. (**C**) Sequencing data from CN21-CHRNG knockout clone. Top trace shows genomic sequence and lower trace shows cDNA sequence. Alignments of WT sequence (top) and the three different alleles with deletions of 25bp, 17bp and 31bp respectively are shown. (**D**) Radioimmunoprecipitation assay showing loss of AChR gamma cell surface expression on CN21-CHRNG knockout clone (N=4). Counts per minute (cpm) before and after background subtraction are shown (background is cpm of empty vials). Error bars represent standard deviation of the mean.

Supplementary methods - CRISPR-Cas9 generation of CN21-CHRNG knockout cell line

CHRNG was disrupted in CN21 cells using standard CRISPR-Cas9 gene editing techniques. Briefly, CN21 cells were transfected using Lipofetamine 2000 with an adapted plasmid derived from pX330-U6-Chimeric_BB-CBh-hSpCas9 (a kind gift from Feng Zhang, Addgene plasmid no. 42330) containing annealed guide oligonucleotides 5'-CACCGACATCCGAGTCTCGTTCCGC-3' and 5'-AAACGCGGAACGAGACTCGGATGTC-3'. Cells were then cloned, genomic DNA was extracted, and the edited region was amplified and sequenced using forward primer 5'-CAGAGAGCTGAGGCACCATGCAT-3' and reverse primer 5'-CTATCCAGACATTGGTGGTG-3'. Clone 3 had undergone non-homologous end joining, resulting in all alleles being out of frame (Fig. S1C). cDNA isolated from this clone also showed the same frameshifts in all alleles (Fig. S1C).

AChR radioimmunoprecipitation assays were then performed on CN21 cell line, TE671 cells and CN21-CHRNG KO cells. Cell surface AChR was radiolabelled with ^{125}I-α-bungaratoxin (^{125}I-α-BuTx), and immunoprecipitated with a CHRNG specific antibody (C9 clone, (1)). Radioactivity was measured using a Wallac Wizard counter, and background counts (measured using empty vials) were then subtracted (Fig. S1D). Cell surface expression of the AChR gamma subunit on clone 3 was undetectable.

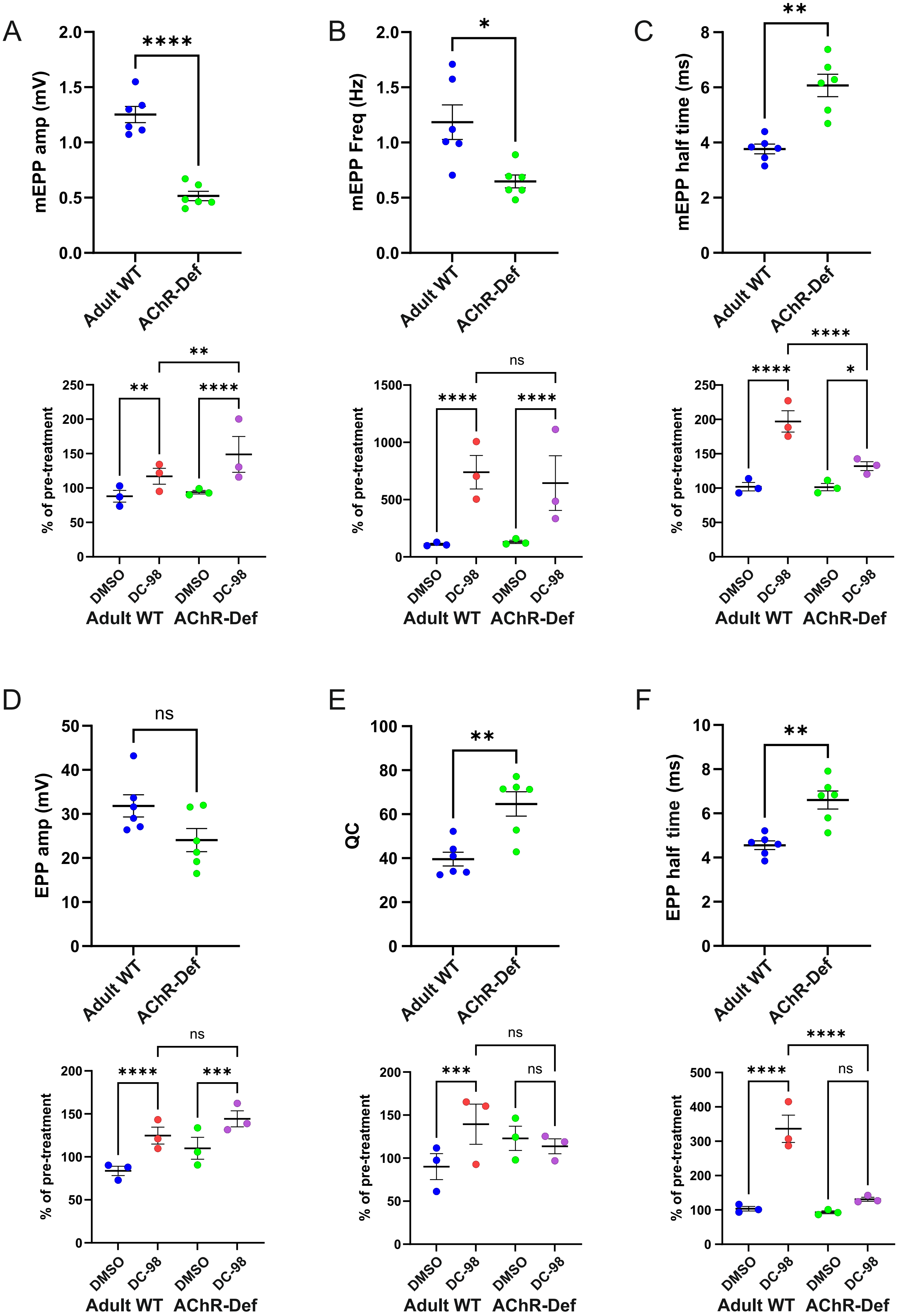

A
mEPP amp (mV)
2.0
1.5
1.0
0.5
0.0
****
Adult WT
AChR-Def
**
****
% of pre-treatment
250
200
150
100
50
0
DMSO
DC-98
DMSO
DC-98
Adult WT AChR-Def
B
mEPP Freq (Hz)
2.0
1.5
1.0
0.5
0.0
*
Adult WT
AChR-Def
ns
****
****
% of pre-treatment
1500
1000
500
0
DMSO
DC-98
DMSO
DC-98
Adult WT AChR-Def
C
mEPP half time (ms)
8
6
4
2
0
**
Adult WT
AChR-Def
****
****
*
% of pre-treatment
250
200
150
100
50
0
DMSO
DC-98
DMSO
DC-98
Adult WT AChR-Def
D
EPP amp (mV)
50
40
30
20
10
0
ns
Adult WT
AChR-Def
ns
****
***
% of pre-treatment
200
150
100
50
0
DMSO
DC-98
DMSO
DC-98
Adult WT AChR-Def
E
QC
100
80
60
40
20
0
**
Adult WT
AChR-Def
ns
***
ns
% of pre-treatment
200
150
100
50
0
DMSO
DC-98
DMSO
DC-98
Adult WT AChR-Def
F
EPP half time (ms)
10
8
6
4
2
0
**
Adult WT
AChR-Def
****
****
ns
% of pre-treatment
500
400
300
200
100
0
DMSO
DC-98
DMSO
DC-98
Adult WT AChR-Def

Fig. S2. **DC-98 modifies various parameters of neurotransmission recorded in diaphragm from Adult WT or AChR-deficiency mice.** In each panel, the upper graph shows a comparison of the untreated adult WT vs AChR-deficiency diaphragm, with each symbol representing the mean of 4-8 fibers from each diaphragm, mean +/- sem for each group is indicated (n=6). The lower graphs of each panel show data from Adult WT or AChR-deficiency preparations with either 0.3% DMSO or 30 μM DC-98 (in the bath) expressed a % of pre-treatment control data from each diaphragm. Each symbol is the mean of 8-14 fibers from each diaphragm, means +/- sem for each group is indicated (n=3). Parameters presented are mEPP amplitude(**A**), mEPP frequency (**B**), mEPP half time (**C**), EPP amplitude (**D**), Quantal content (QC, **E**), EPP half time (**F**).

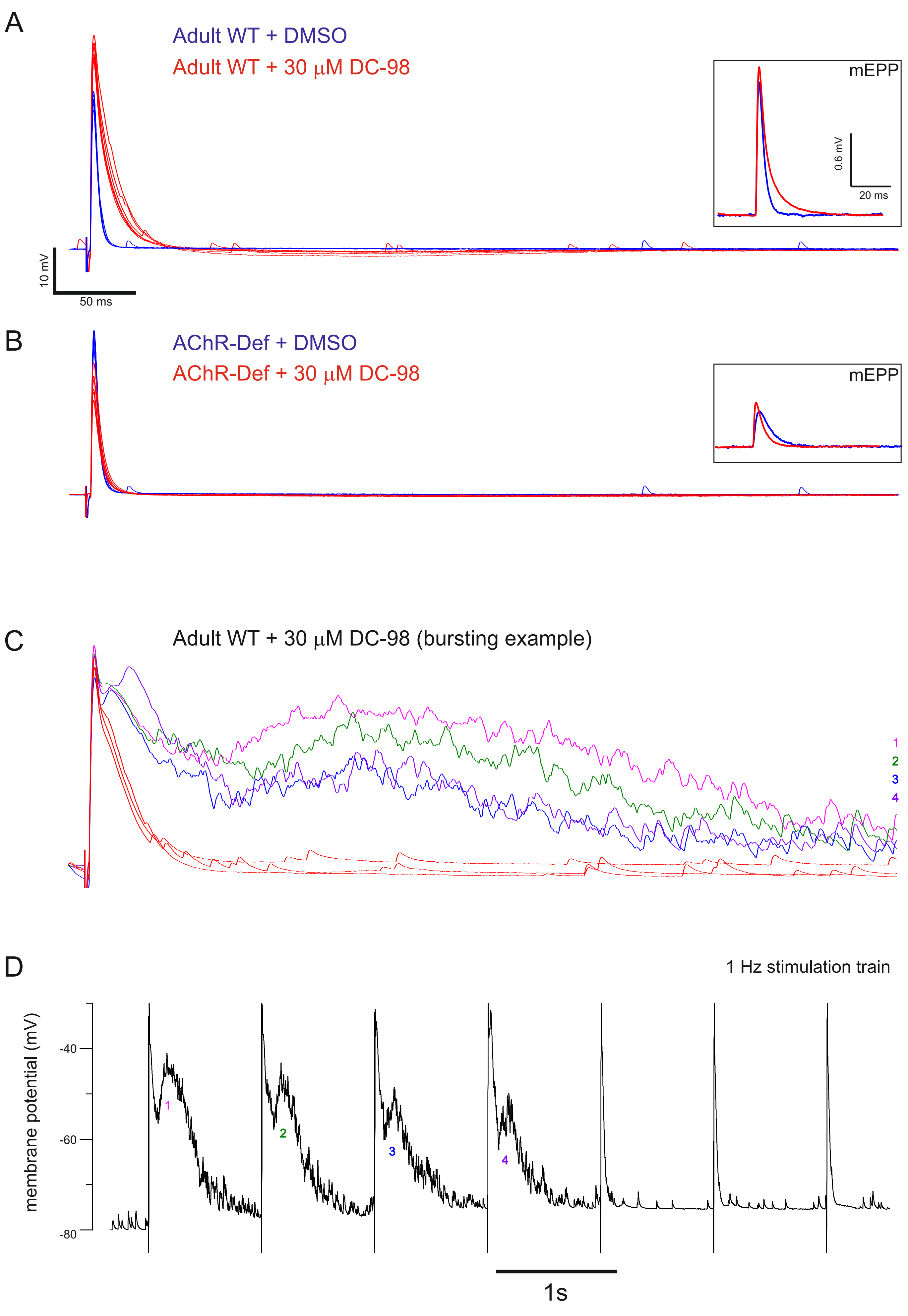

A
Adult WT + DMSO
Adult WT + 30 μM DC-98
mEPP
0.6 mV
20 ms
10 mV
50 ms
B
AChR-Def + DMSO
AChR-Def + 30 μM DC-98
mEPP
C
Adult WT + 30 μM DC-98 (bursting example)
1
2
3
4
D
1 Hz stimulation train
membrane potential (mV)
-40
-60
-80
1
2
3
4
1s

Fig S3. Example traces of EPP and mEPP recordings from Adult WT and AChR-deficiency mouse diaphragms. (A) shows example traces of EPP recordings from Adult WT diaphragm with either 0.3% DMSO (blue) or 30 μM DC-98 (red), inset shows mEPP recordings. (B) shows example traces of EPP recordings from AChR-deficiency diaphragm with either 0.3% DMSO (blue) or 30 μM DC-98 (red), inset shows mEPP recordings. (C) shows example of recording from Adult WT diaphragm with post-EPP mEPP disorganized release, 1st 4 stimuli in the 1 Hz train are individually coloured to indicate sequence, for reference to panel (D). (D) shows continuous trace of recordings from (C) at slower time base, to indicate reversion to normal EPP after 4 stimuli (red in panel (C).

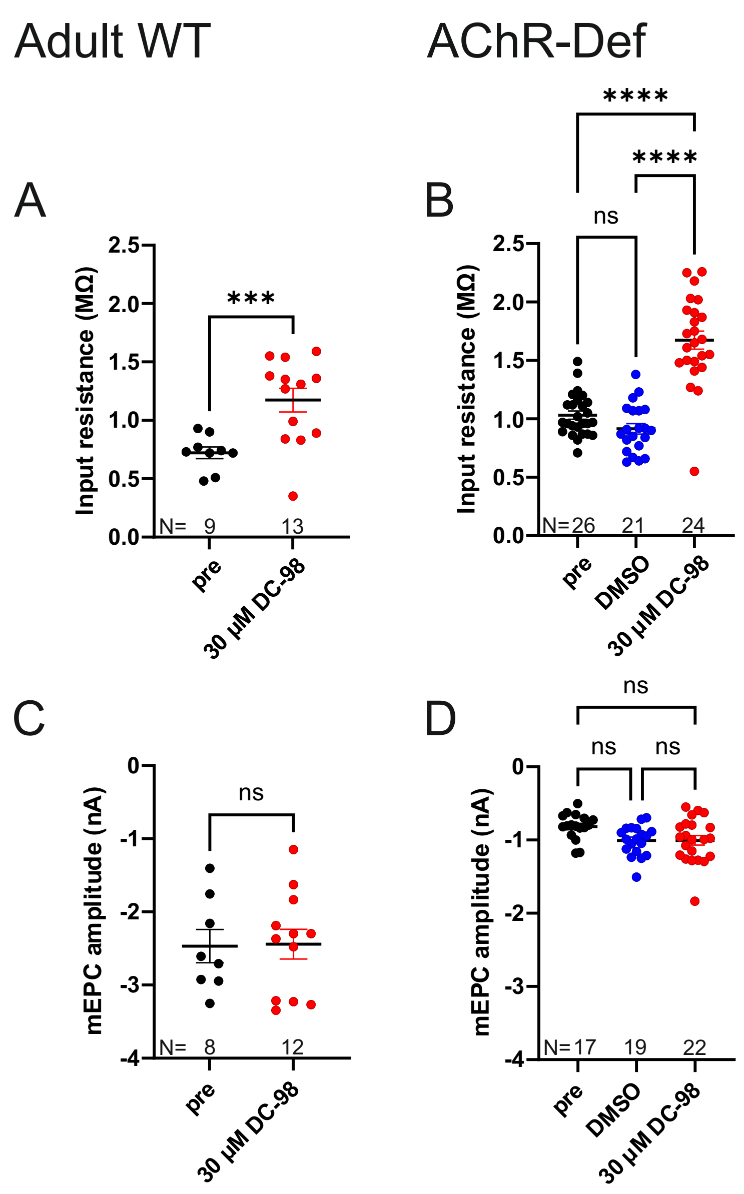

Fig S4. Effects of DC-98 on membrane input resistance and mEPC amplitude. The effect of 30 µM DC-98 on input resistance (A) and mEPC amplitude (C) in adult WT phrenic nerve/diaphragm preparations, with mean ± sem shown, each data point is from an individual fiber. The effect of 30 µM DC-98 and DMSO on input resistance (B) and mEPC amplitude (D) in AChR deficiency phrenic nerve/diaphragm preparations, with mean ± sem shown, each data point is from an individual fiber. Comparisons for adult WT were by unpaired t-tests (N=9 &13 and 8 &12), whilst comparisons in AChR-deficiency data were by ANOVA and t-tests corrected for multiple comparisons (N= 26, 21& 24 and 17, 19 & 22).

1.P. J. Whiting, A. Vincent, M. Schluep, J. Newsom-Davis, Monoclonal antibodies that distinguish between normal and denervated human acetylcholine receptor. J Neuroimmunol 11, 223-235 (1986).
